# Assessing the cost-effectiveness of contraceptive methods from a health provider perspective: case study of Kiambu County Hospital, Kenya

**DOI:** 10.1186/s12978-021-01308-3

**Published:** 2022-01-17

**Authors:** James Kiragu Ngacha, Richard Ayah

**Affiliations:** Department of Public & Global Health, University of Nairobi, Nairobi, Kenya

**Keywords:** Contraceptive methods, Contraceptive prevalence rate, Cost-effectiveness, Couple year protection

## Abstract

**Background:**

Kenya’s contraceptive prevalence rate at 53% is low, with wide disparity among the 47 counties that make up the country (2–76%). Significant financial investment is required to maintain this level of contraceptive use and increase it to levels seen in more developed countries. This is in the context of a growing population, declining donor funding, limited fiscal space and competing health challenges. Studies have shown that long-term contraceptive methods are more cost-effective than short-term methods. However, it is unclear if this applies in Sub-Saharan Africa; with limited financial resources, lower social economic status among users, and publicly managed commodity supply chains, in vertical programs largely dependent on donor funding. This study assessed the cost-effectiveness of contraceptive methods used in Kenya.

**Methods:**

A cross-sectional study was undertaken in a county referral hospital in mid-2018. Purposive sampling of 5 family planning clinic providers and systematic sampling of 15 service delivery sessions per method was done. Questionnaire aided interviews were done to determine inputs required to provide services and direct observation to measure time taken to provide each method. Cost per method was determined using activity based costing, effectiveness via couple year protection conversion factors, and cost-effectiveness was expressed as cost per couple year protection.

**Results:**

The intra-uterine copper device was most cost-effective at 4.87 US dollars per couple year protection followed by the 2-Rod Implant at 6.36, the 1-Rod Implant at 9.50, DMPA at 23.68, while the combined oral contraceptive pills were least cost-effective at 38.60 US dollars per couple year protection. Long-term methods attracted a higher initial cost of service delivery when compared to short-term methods.

**Conclusion:**

Long-term contraceptive methods are more cost-effective. As such, investing in long-term contraceptives would save costs despite higher initial cost of service delivery. It is recommended, therefore, that Sub-Saharan Africa countries allocate more domestic financial resources towards availability of contraceptive services, preferably with multi-year planning and budget commitment. The resources should be invested in a wide range of interventions shown to increase uptake of long-term methods, including reduction of cost barriers for the younger population, thereby increasing contraceptive prevalence rates.

## Plain language summary

The proportion of women of reproductive age using a contraceptive method in Kenya, at 53% is low. More funding is required to ensure women who need contraceptives have access and continue using them. Previous research has shown that contraceptives that protect a woman from getting pregnant for an extended period utilize less resources. This study assessed the potential of contraceptives used in Kenya to produce results at less cost.

This study was undertaken in a sub-national referral hospital in mid-2018. A sample of 5 family planning clinic providers were interviewed while 15 contraceptive service sessions per method were observed. The interviews were conducted to determine the supplies required to offer services. Observation was used to measure time taken to provide each contraceptive method. The cost associated with providing each contraceptive per year was determined using activity based costing. Effectiveness of each method was determined using the rate of protection from pregnancy during one-year. Cost-effectiveness was the money utilized to achieve protection from pregnancy in one year of use.

The Copper-T coil was found to achieve greater results at a lesser cost of 4.87 US dollars for one year of protection from pregnancy, followed by the 2-Rod Implant at US dollars 6.36, the 1-Rod Implant at 9.50, DMPA at 23.68, and The Pill at 38.60 US dollars.

Contraceptive methods that protect a woman from getting pregnant for a longer period utilize less resources. Investing in such would save costs despite requiring higher initial cost of service provision.

## Introduction

### Background

In 2017, it was estimated that of the 1.6 billion women of reproductive age in developing countries, about 885 million needed contraception [1]. The unmet need for contraception in developing countries stood at about 214 million women with the unmet need being highest in Sub-Sahara Africa at 21% [1]. According to the Kenya Demographic and Health Survey (KDHS) 2014/15, Kenya had a contraceptive prevalence rate (CPR) of 58% for all methods (and 53% for modern methods—mCPR) among married women, up from 46% in 2009 [2]. The 2019 estimated mCPR among married women was 61.4 [3]. The KDHS 2014/15 put the unmet need for contraception in Kenya at 18% for married women (26% for all women of reproductive age) down from 26% (married women) in 2009 [2]; 2019 estimate is 16.9% [3]. The 2014/15 mCPR was dominated by Depot Medroxy-Progesterone Acetate (DMPA) contributing 26%, followed by implants at 10%. Pills contributed 8% while intrauterine copper devices (IUCDs) and sterilization contributed about 3% each. The rest was contributed by condoms and traditional methods among others [2]. Kiambu County, one of the 47 counties in Kenya, had a CPR that is higher than the national average at 74% for all methods and 68% for modern methods among married women [2]. In this county, DMPA contributed 22%, followed by pills at 19%. Implants contributed 12%, IUCDs contributed 9%, female sterilization contributed 3%, while condoms and non-modern methods contributed the rest [2].

Contraceptive programmes are some of the most cost-effective tools available for tackling public health challenges of our day [4, 5], and also contribute to economic growth and development [1, 6]. Contraceptives are known to significantly reduce maternal and child mortality, over and above directly reducing the number of unwanted pregnancies and unsafe abortions [1]. Contraceptives have also been shown to contribute positively to factors of economic growth and development especially in relation to women and children [6]. From an economic point of view, the use of contraceptives is clearly more beneficial than failure to use; with cost-benefit ratios of 1:9 in UK [4], 1:7 in USA [5], 1:2 in Sierra Leone [7], and 1:4 in Kenya [8] among other studies that show similar trends [9–12]. However, questions persist regarding which methods are more cost-effective.

Long-acting contraceptive methods appear to be more cost-effective than short-acting methods from all perspectives [13–17]. This can be explained by long-acting methods being more efficacious [18], that is, have a lower failure rate when used correctly and consistently. Therefore, each service results into higher benefit when viewed from health provider perspective and less visits to the health facilities for the same level of effectiveness from clients’ perspective. Further, long-acting methods are less dependent on user compliance and their efficacy is close to or similar to typical use effectiveness [18]. There is, therefore, less cost associated with their failure and consequences of failure [13–15]. From clients’ perspective, this means less costs associated with unwanted pregnancies, and reduced workload in form of antenatal, postnatal and peri-natal healthcare needs from provider’s and health system’s perspectives. From the society perspective, less contraceptive failure results into better population management and associated economic benefits. Published data shows that implants are the most efficacious method with about 0.05 unintended pregnancies per 100 women per year; IUCDs 0.6, while DMPA and Combined Oral Contraceptive Pills (CoCs) have a failure rate of 0.3 [18]. This is in contrast to male condoms that have a failure rate of 2 unintended pregnancies per 100 women per year, 5 for female condoms, 6 for cycle beads, 18 for spermicides, and use of no method would result into about 85 pregnancies [18].

A number of studies have shown some short-term methods dominating their long-term counterparts in cost-effectiveness for the entire or part of the study period [13, 19–21]. This could be explained by some studies having only considered fixed costs [21], or considered a period of less than 2 years [13, 19], or differing market dynamics (context) for example cost of commodities [20]. Some studies showed implants as the least cost-effective method [19–21]. This could be explained by differing market dynamics and high cost of implants at the time of the studies.

Several factors affect real-world effectiveness of contraceptives. Short-term methods are not only less efficacious than long-term methods, but also their effectiveness (CoCs and DMPA) is highly dependent on correct and consistent use [14, 18]. Although user compliance is not a major factor in determining effectiveness of long-acting reversible contraceptives (LARCs), continuation/discontinuation of use, which is user-dependent, has an impact on effectiveness. The mean 1-year continuation rate of etonogestrel implants, for example, was higher among women in middle-income countries during clinical trials than in developed countries [22]. Subsequent studies showed higher continuation rates in lower income countries (98% in Nigeria) [23] than in developed countries (87% in USA [24] and 75% in the UK) [25]. Drug interactions and general health status of users also determine effectiveness of contraceptives [18]. Various drugs especially protease inhibitors used as part of Antiretroviral Therapy (ART) in treatment and prophylaxis of HIV/AIDS are known to reduce effectiveness of hormonal contraceptive methods [18]. Age of users affect effectiveness of some contraceptive methods with younger users having higher number of pregnancies prevented in the case of sterilization [13]. Frequency of sexual intercourse determines effectiveness of methods that are dependent on sexual activity for example diaphragm, spermicides and condoms [26]. From the above, it is clear that contextual factors have an effect on effectiveness of various contraceptive methods (both long and short acting methods) due to varying socio-economic status of users, prevalence of infectious disease and lifestyle factors.

Various studies evaluated cost from different perspectives—clients, health system and wider society perspectives. From the clients’ perspective, costs included user fee and transport, consequences of method failure as well intangible costs like psychological impact of methods failure [27, 28]. From health system perspective, costs were either fixed (capital and overheads at facility and health system management levels) or recurrent (labour, commodities and equipment) [17, 20, 21] or determined via cost modeling [13]. Societal perspective would include all the above costs plus opportunity cost of providing the services and costs to the society due methods failure [14, 27]. The choice of cost perspective depended on study objectives, resources (financial, time and human), and availability of data.

In summary, long-term contraceptive methods are expected to attract higher cost of service delivery (from provider perspective) when compared to short-term methods [13, 14] and more cost-effective in the long run. This can be attributed to higher cost of commodities especially for implants [29], cost of labour, training, and equipment required to provide the long-term methods [13, 30]. Due to lower cost of service delivery, short-term methods are therefore likely to be more cost-effective in 1 to 2 year horizon [13–15]. ‘Decisions of value’ that would affect the cost of providing healthcare services are influenced by a wide range of ‘inner and outer context’ [31] that range from information, organizational culture, governance, economic and political conditions [32]. The cost associated with each method is, therefore, expected to vary with cost perspectives, but also, from country to country, region to region of each country, and even from facility to facility given their unique contextual factors. Irrespective of the cost-effectiveness profile of the available methods, informed clients’ choice is paramount and family planning programs should always have a wide range of methods in all service delivery points.

### Research problem

Kenya’s mCPR at 53% [2] (2019 estimate is 61% [3]) among married women remains low. A lot of investment is required to sustain this CPR in the face of growing population, and increase it to the levels seen in more developed countries (77% in N. Europe, 82% in Eastern Asia, and 75% in N. America) [33]. This is in an environment of reducing donor funding that has been the backbone of the FP program in Kenya and inadequate local resources allocation by both the national and county governments. Efficient use of resources is, therefore, critical.

Several studies have shown that long-acting contraceptive methods are more cost-effective than short-term methods [13–15]. However, most of these studies have been carried out in more developed countries where healthcare and contraceptive programmes have differing dynamics compared to Kenya and therefore Kiambu County. Some of the possible contextual differences include: involvement of the private sector, social economic status of providers and clients, health insurance coverage (including other forms of risk pooling), commodities’ supply chain management, and levels of unmet need. Unique country (and in this case county) specific data is critical for prudent decision making. This study was, therefore, designed to assess cost-effectiveness of various contraceptive methods in the context of Kiambu County.

### Research question and objectives

This study sort to answer the question; “What is the cost-effectiveness of contraceptive methods available in Kenya?” The objective of this study was to determine the cost-effectiveness of contraceptive methods used in Kenya.

## Methods

### Study design

This research was a cross-sectional study to determine the cost-effectiveness of contraceptive methods offered in Kiambu County Hospital in Kenya. This was a full economic evaluation encompassing assessment of cost, effectiveness and several alternative courses of action.

### Study site and population

Kiambu County Hospital was chosen because of convenience (about 15 kilometres from Nairobi City) and the County has a higher mCPR (68%) than the national average of 53%, and higher contribution of long-term methods than the national average [2]. Kiambu County Hospital is a Public Hospital and one of the 45 Level 4 (Kenya Essential Package for Health classification) hospitals in Kenya—36 of the hospitals (80%) being private (for profit and not for profit) while 9 (20%) are public (owned and managed by the Kiambu County Government) [34]. In 2016/17 financial year, Kiambu County Hospital had a total work load of 355,910 (outpatient visits plus number of bed days) [35]. The study population consisted of the healthcare providers who offer family planning services in the hospital.

### Selection of alternatives

The following contraceptive methods were selected for this study because they contributed 89% of mCPR in Kenya and 91% of mCPR among married couples in Kiambu County [2]:

i. Intra-uterine copper devices (IUCDs)—Copper T® 380
ii. Contraceptive implants;
  - 2-Rods Contraceptive Implants, each containing 75 mg levonorgestrel—Jadelle®
  - 1-Rod Contraceptive Implants, each containing 68 mg etonogestrel—Implanon NXT^®^
iii. Depot Medroxy-Progesterone Acetate (DMPA) 150 mg, Intramuscular
iv. Combine Oral Contraceptive Pills (CoC—Pills)—a cycle of 28 pills having 21 pills each containing Ethinylestradiol 30 mcg, and Levonogestrel 150 mcg and an extra 7 Ferrous Sulfate pills that are supplied in a pack of 3 cycles under an MoH, Kenya owned brand called ‘Chaguo Langu’

### Cost of providing each method (dependent variables)—sample, sampling and data collection

**Fig. 1:**
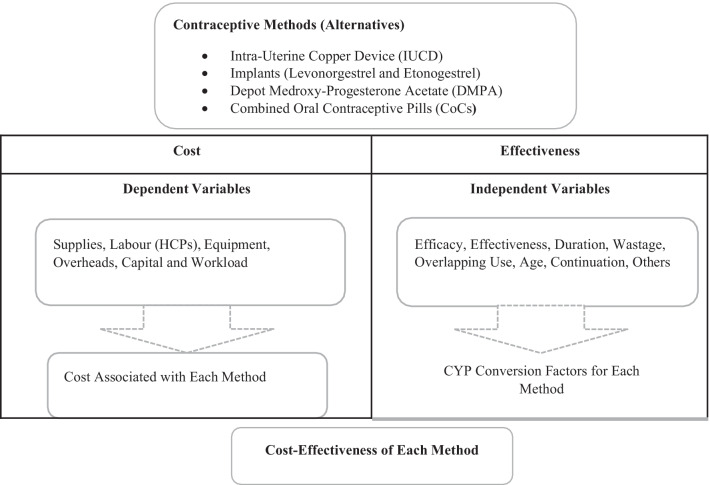
Conceptual framework

Cost was estimated through Activity Based Costing model [36, 37] as shown in the study’s conceptual framework (Fig. 1).

Inputs required to provide each method were classified into; supplies, labour, equipment overheads, and capital. A purposeful sampling of all healthcare providers manning the FP clinic on day one of the study was done. The study was carried out between July and October 2018. Questionnaire aided interviews (5 respondents) was carried out to determine supplies, equipment, capital, and overheads required to provide IUCDs; implants; DMPA and CoC Pills, and number of routine follow-up visits per method. This was followed by in-depth interviews (2 respondents) to clarify the client’s pathway and exact identity of inputs identified above; for example, the brand and pack size of glove, the brand name and other specifications of the Blood Pressure machine used and so forth for the purposes of costing. Information from the in-depth interviews and questionnaires was validated through procurement records and observation of available supplies, equipment and capital in the facility. The hospital workload for 2017/17 was used to distribute cost of capital, shared equipment, and overheads among the studied methods. Time taken to provide each method was used to estimate labour cost and was collected via direct observation of at least the first 15 service deliver sessions per method, selected via systematic sampling. The time at entering and leaving the service delivery room was noted on pre-designed tally sheets.

Sample size of the number of services to be observed was calculated as follows [38]; n = ((Z 1/2 α)^2^ x SD^2^ ) ÷ d^2^ : where Z 1/2 α = 1.96 (α = 5%), expected standard deviation = SD (5 min), and acceptable level of error = d (3 min). The slowest method to provide was assumed to take an average of 40 min

Secondary data was collected from various sources as shown in Table 1.

**Table 1:** Secondary data and their sources

Data	Source
Staff Establishment for Kiambu County Hospital	Hospital Admin Records—2016/17 FY
Posting to MCH Clinic and Duty Roster	Hospital Admin Records—Duty Roster (August 2018—MCH Clinic)
Salary scale for various job groups	County Admin Records (data from Nairobi County was used—2017 salary scale)
Hospital expenditure for the 2015/17 financial year	Hospital admin records—Accounts report of 2016/17 FY
Space used for MCH (including FP services) and Central Sterile Services Department (CSSD)	Maintenance records—2018
Workload for 2016/17 financial year	• DHIS 2 [35] and MoH 717 (Service workload—2016/17 FY) • MoH 711 (FP)—2016/17 FY • CSSD records—2016/17 FY
Equipment and capital	• Hospital Admin Records—Accounts Report (2016/17 FY) • Maintenance Records—2018
Prices of supplies, equipment and FP commodities	• KEMSA Pricelist (KEMSA, 2018) • UNFPA Pricelist (UNFPA, 2018) • Private sector vendors—trade prices 2018 • FP Quantification and forecasting booklet (2018–2020 technical report) [39] • Hospital Admin (procurement) records—2018
Useful life of various equipment and capital used to provide contraceptive methods	• WHO Website (CHOICE)— [40] • University of California—equipment useful life table for depreciation (revised to reflect 1998 AHA schedule) • Kenya Insurance Guideline on useful life for compensation purposes (2004)

### Effectiveness of each method (independent variables)

Effectiveness was estimated from Couple Years Protection (CYP) conversion factor associated with each method—that translates services into common impact (prevention of pregnancy). CYP conversion factors take into account the efficacy of each method, duration of use, effectiveness, coital frequency for coitus dependent methods, wastage (for self-administered methods), misreporting (accounts for data quality), age (for sterilization), consistency of use, non-contraceptive use (for condoms), and overlapping coverage (use of more than one method at a time) that influence effectiveness of contraceptive services [26]. This measure of effectiveness is easy to use and requires the number of services offered or volume of commodities distributed as the only input. Data used to construct conversion factors often include data from developing countries on factors that affect effectiveness of contraceptive services [26]. A study conducted in Iran used Adjusted CYP conversion factors as the measure for effectiveness of various methods [20] and quoted its robustness and ease of use as the reasons for choosing it. A study conducted in Kenya [21] also used CYP as the measure of effectiveness. In summary, the use of CYP conversion factors as a measure of effectiveness is simple, uses service data as the only input, considers the context of developing countries and is appropriate for use in studies like this one. Conversion factors used in this study were obtained from USAID website [41].

## Analysis

Data from primary or secondary sources was entered in a pre-designed Microsoft Excel sheet, cleaned and processed for further analysis.

### Calculations

The study utilized an Activity Based Costing model to determine the cost of providing the various contraceptive services to clients [36, 37]. Calculations were done using Microsoft Excel sheets. The following steps were followed to estimate cost: identification of inputs needed per activity and per method, quantification of inputs per method, costing of inputs per method, computation of cost per method, and finally calculation of cost per couple years of protection.

Price was used to estimate the cost of inputs. Cost of supplies was determined from the average prices by public sector suppliers (for a few products that were not available through public sector supply chain, private sector prices were used) at the time of the study. Cost of FP commodities supplied to the hospital as donations was estimated from average price to the donors plus 10% cost freight, customs clearance, storage and in-country distribution. Cost of labour was determined from time taken to provide each method, job groups of the providers, and average salary of each job group (as set by salaries and Remuneration Commission of Kenya). Cost of equipment was determined from the prices of procuring new equipment at the time of the study, amortized as per their useful life years and then distributed among various methods using the workload. Cost of overheads was determined from 2016/17 FY facility expenditure and hospital workload. Capital cost was determined from space utilized and amortized cost of putting up new building, and cost of procuring new furniture and other required installations at the time of study (amortized as per their useful life years) distributed to each method using workload.

Cost of each method was then divided by CYP conversation factor for each method that as available on USAID website [41] accessed on 5th August 2017 to determine cost-effectiveness of each method. The various methods were then ranked according to their cost-effectiveness with the method showing the lowest cost per CYP being deemed the most cost-effective as shown in Table 2.

**Table 2:**
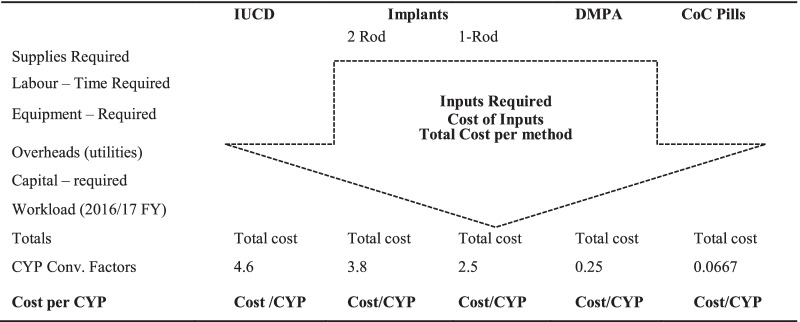
Inputs required and contraceptive methods

### Ethical considerations

Ethical approval was obtained from Kenyatta National Hospital/University of Nairobi Ethical Review Committee (KNH/UoN-ERCA210). Further approval was received from the Kiambu County Health Research Development Unit and the Kiambu County Hospital management before commencement of the study.

## Results

### Inputs required to provide services

The IUCD was found to be the most cost-effective method at 4.87 USD (CI 4.16–5.60) per CYP (exchange rate 103KeS to the USD), followed by the 2-Rod Implant at 6.36 (CI 5.85–6.87), the 1-Rod Implant at 9.50 (8.72–10.28), DMPA at 23.68 (CI 21.39–24.86), and CoC Pills at 38.60 (CI 0.40–46.82) was the least cost-effective. The cost-effectiveness ratio when compared to IUCD was as follows; 2-Rod Implant 1.3, 1-Rod Implant 1.95, DMPA 4.86, and COC Pills 7.92. The 2-Rods Implant attracted the highest initial cost of services delivery per client at 24.19 (70% recurrent i.e. supplies and equipment), followed by 1-Rod Implant at 23.78 (70% recurrent), while IUCD was the least expensive long-term method to provide at 22.41 (46% recurrent) per client. On the other hand, short-term methods attracted less initial cost of service delivery with DMPA costing 5.91 (55% recurrent) and CoC Pill at 2.57(50% recurrent) per client. On average, labour, supplies and overheads were the main cost drivers accounting for 92% of the cost, each contributing about 30% of the total cost. Cost of FP commodities alone contributed 40% for the 2 Rod Implant, 42% for the 1-Rod Implant, 2.5% for the IUCD, 19% for DMPA and 10% for CoC Pills.

Seventeen (17) individual supplies items were needed to provide services of the contraceptive methods studied. The CoC pills required only the FP commodity while DMPA required syringe and needle and alcohol swab as well. The IUCD required chlorhexidine, sterilized gloves, surgical masks and gauze swabs while the implants required lidocaine, syringe and needle, povidone iodine solution, gauze swabs, adhesive plaster, sterile gloves, non-sterile gloves and pressure bandage. All the clients needed triage equipment notably blood pressure (BP) machine, thermometer, weighing scale and stethoscope. Long-term methods required more of the 24 pieces of equipment identified due to sterile/aseptic procedures required to insert and remove the methods. The IUCD and implants required the most provider time among long-term methods (IUCD 46 min (n = 33, CI 28–63), 2-Rod Implant 35 min (n = 23, CI 25–46) and 1-Rod Implant 29 min (n = 39, CI 19–38). On the other hand, among the short-term methods, DMPA required 6.50 min (n = 27, CI 5.5–7.5) and an average of 2 cycles of CoC Pills 7.10 min (n = 19 CI 3.5–10.8). Implants and IUCD attracted more overhead costs than the short-term methods since they required sterilization of insertion and removal equipment. The capital cost was evenly distributed (per visits) among all the methods as shown in Table 3.

**Table 3:** Inputs required per method and contraceptive services (N-149)

Dependent Variable	Method	Insertion/provision	Check-ups	Removal	Total cost	Confidence interval
		USD	USD	USD	USD	
Supplies	Implants-Rod 2	9.88	0.02	0.68	10.58	–
	Implants-Rod 1	10.03	0.02	0.68	10.73	–
	IUCD	1.17	0.25	0.42	1.84	–
	DMPA	1.12	–	–	1.12	–
	CoC Pills	0.24	–	–	0.24	–
Labour	Implants-Rod 2	2.80	0.45	3.19	6.44	[4.5−8.38]
	Implants-Rod 1	2.58	0.42	2.87	5.87	[3.92−7.83]
	IUCD	3.45	2.11	3.02	8.57	[5.25−12.01]
	DMPA	2.13	–	–	2.13	[1.80−2.46]
	CoC Pills	1.06	–	–	1.06	[0.51−1.60]
Equipment	Implants-Rod 2	0.58	1.09	0.58	1.28	–
	Implants-Rod 1	0.58	0.12	0.58	1.28	–
	IUCD	0.52	0.42	0.52	1.47	–
	DMPA	0.17	–	–	0.17	–
	CoC Pills	0.03	–	–	0.03	–
Overheads	Implants-Rod 2	2.40	0.48	2.40	5.27	–
	Implants-Rod 1	2.40	0.48	2.40	5.27	–
	IUCD	3.46	2.77	3.46	9.68	–
	DMPA	2.22	–	–	2.22	–
	CoC Pills	1.11	–	–	1.11	–
Capital	Implants-Rod 2	0.28	0.06	0.28	0.62	–
	Implants-Rod 1	0.28	0.06	0.28	0.62	–
	IUCD	0.30	0.24	0.30	0.84	–
	DMPA	0.27	–	–	0.27	–
	CoC Pills	0.14	–	–	0.14	–

## Discussion and conclusion

The results of this study were generally consistent with findings of similar studies [13–17, 28] where long-term methods dominated short-term methods; with the IUCD attracting lowest cost per CYP, followed by the 2-Rod Implant (1.3 times more than IUCD), the 1-Rod Implant (1.95 times), DMPA (4.86 times), and finally CoC Pills (7.92 times). The difference with findings of deviating studies may be attributed to differing market dynamics (contextual factors such as price of commodities especially implants in the country the studies were carried out [19, 20] or time of the study [21] ) as well as a drastic reduction in the price of implants in FP 2020 Countries in 2013 [42, 43]. Some studies showed a number of short-term methods dominating their long-term counterparts, for entire time of the study or part of the study period [13, 19–21]which can be explained by failure to consider fixed costs [21] or considering short period (less than 2 years) [13, 19]. In our study, fixed costs accounted for 38% on average—54% for IUCD, 50% for CoC Pills, 45% for DMPA and 30% for implants. In agreement with other studies, we found that long-term methods attracted higher initial cost of service delivery when compared to short term methods. The 2-Rods Implant attracted the highest cost of services delivery 24.19 USD, followed by 1-Rod Implant at 23.78, IUCD at 22.41, DMPA at 5.91 and CoC Pill at 2.57 per client. This can be explained by higher cost of procuring commodities [13, 17, 20, 21], higher cost of labour (HCP time) [13, 17, 20, 21], and cost of equipment and supplies [13, 44] required to provide long-term methods. Over and above this, long-term methods attract additional cost to the health system in form of training the HCP [42, 44] to offer services. We, therefore, can conclude that long-term contraceptive methods are more cost-effective than short-term methods, but require more initial investment in terms of commodities, equipment, labour, and training of service providers.

These results should be interpreted with caution given the following limitations. First, this was a case study carried out only in one hospital and reflects the unique context within the hospital, for example, the skills mix of the healthcare provider (HCP) manning the FP clinic. Second, only the cost of labour was subjected to statistical analysis. Other costs were based on secondary data and actual measurements, and workload was used to distribute cost among the methods. Changes in the hospital workload and that of various contraceptive methods could affect the results [20]. Third, the study only considered cost from provider perspective. Costs from other perspectives could have changed the total cost per method without changing the cost-effectiveness pattern observed since the same fundamentals (less utilization of services per unit of effectiveness and lower failure rate) drive cost-effectiveness. Several studies from client and societal perspectives shows the same pattern; long-acting methods are more cost effective [15, 28]. Fourth, the study did not include costs associated with contraceptive method failure and management of adverse effects. These costs would most likely enhance cost-effectiveness seen among long-term methods since they have less failure rates.

Increasing the use of long-term contraceptive methods involves investing both in the supply side (training, support supervision and mentoring, commodity security, multiple service delivery models (including outreaches) and demand generation for FP services [43–45]; while putting the clients’ right to make informed choice at the centre. This kind of investment requires long-term planning with possibility of multi-year budgets. Long-term planning and budgeting are currently possible with donor funded vertical FP programs given multi-year commitment by donors. Donor funding has been the main source of financial resources for FP programs in SSA since their inception [46, 47]. However, this has been on decline in the recent years and it is likely to reduce even more with the reintroduction of the Global Gag Rule in 2017 with expanded interpretation [48]. For Kenya, the decline is likely to be even more pronounced due to attainment of lower-middle income status since 2014 [49]. There is need to mobilize more domestic funding to ensure adequate service delivery and long-term sustainability [47, 50]. Unfortunately, domestic resources which are based on government annual budgets may not promote long-term planning. Such annual budgets run the risk of being based on costs of providing various contraceptive methods other than their long-term cost effectiveness.

Some African countries, including Kenya, have budgetary allocation (though inadequate) for FP programs including for procurement of FP commodities [46, 47]. Whether the budgets are ring-fenced for FP commodities and services is subject for a separate discussion. For Kenya, utilization of the inadequate domestic funds is complicated by devolution of health services where policy direction is a function of the national government while service delivery is at the county level (sub-national level) [47, 50]. Some counties in Kenya have FP budgets [50, 51] but unfortunately, the budgets are inadequate and much of the FP related expenditure at the county level only cater for FP commodities and service delivery [51] other than the comprehensive investment that has been shown to increase utilization of long-term contraceptive methods. Having up-to-date FP Costed Implementation Plans (CIP) at both the Central Government and each of the 47 counties could be one of the ways to ensure long-term planning as well as coordinated investment to increase use of long-term methods. The central government has a 2017-20 FP CIP [52] but only a few counties have FP CIPs [51].

Whereas cost of contraceptives has been identified as a barrier to accessing FP services [53], (especially LARCs [54, 55]) by all women, it is one of the most important barriers to youth and adolescent access to long-term contraceptive methods [30, 56–59]. Removing cost barriers among other investment was found to increase uptake of long-term methods [30] by youth and adolescents; which is a growing demography in almost all developing countries including Kenya. FP services in FP 2020 countries is heavily subsidized through vertical donor funded programs [46, 47, 60]. With declining donor funding, domestic sources of financial resources would again be required to continue subsidizing FP services. Innovative solutions within Universal Health Coverage framework where financially vulnerable groups like the youth and adolescent access services for free could be one solution that removes cost barriers to accessing long-term contraceptive methods. This would have the overall effect of increasing the proportion of CPR contributed by long-term methods—since young people form a very large proportion of the populations in Africa (20% of all global youth (15-24 years), which is expected to rise to 42% by 2030) [61].

## Recommendations

In the current environment of declining donor funding, it is important for each country in Sub-Saharan Africa to allocate adequate financial resources to ensure continued availability of FP services and sufficiently invest in long-term methods. Adequate resource allocation is especially important for long-term methods that not only attract a higher initial cost of service delivery, but also require further investments in form of training services providers and procurement of equipment. Budget commitments should be multi-year in nature to enable long-term planning based on long-term cost saving other than initial cost of providing the services and capacity building. The budgetary allocation should cover all the investments that have been shown to increase use of long-term contraceptive methods such as; training of service providers, multiple and responsive service delivery options, commodity security, equipping facilities and demand creation. Funds for procuring FP commodities need to be in their specific budget lines. This is because FP commodities account for a very significant proportion of the overall cost of providing FP services but also form a very critical input to the services.

Resource allocation at the national level alone may unfortunately not result into availability of FP services or increased use of long-term methods. Adequate resource allocation at the sub-national levels for example local municipal governments that are responsible for health service delivery is also vital. Unlike in the current donor funded vertical FP programs, it is difficult to have domestic funds allocated to sub-national authorities that can only be used to deliver FP services. Unless local authorities and health care managers at the local level prioritize FP, services could continue to be inadequate at the local level despite resources commitment at the national level because of the following reasons. First: in some regions, the demand for FP services may be low and, local authorities and healthcare managers may allocate resources to more pressing healthcare needs. Second: local authorities are less likely to feel the pressure to meet international commitments to the same extent as national government. For example, many countries in SSA have made commitments to improve access to family planning services through the FP 2020 movement. The national governments may align their budgets to such global and other regional commitments only for the funds to be diverted at the local level. Third; local authorities and healthcare managers’ interests may not align with family planning objectives since higher populations may often translate to higher resource allocation to their regions.

Lastly, it is important for the current subsidies that are inbuilt in most FP programs in SSA to continue or where possible be enhanced, even in the face of declining donor funding, economic challenges and competing priorities. Justice, as an ethical principle clearly support subsidizing FP services given that there are many positive externalities associated with use of contraceptives. Women (even those of high economic status and can afford) should not fully meet the cost of using contraceptives when the entire society benefit from the use. The principle of equity, both as a desirable goal of the health system and an ethical principle also support subsidies in the Family Planning services. Women the world over and even more so in the SSA are considered to be less economically endowed when compared to men while they consume the highest proportion of contraceptive services. Further, youth and adolescents are considered economically vulnerable and removing cost barriers for this demography has been associated with improved uptake of FP services. Continuing to make FP commodities available, free of charge, to all service delivery points (both public and private) is one way that would ensure subsidies continue in an equitable way—where those of lower economic status access service without any user fee (or minimal user fee at public facilities to cover other costs of service delivery) while those of higher economic status pay more user fee in private facilities. Market segmentation hence total market approach to providing FP services would be achieved without putting undue burden on women or incurring huge administrative cost of trying to keep free commodities in the public facilities only. Removing all cost barriers for the most economically vulnerable, especially youth and adolescents, for example through vouchers covering transport cost and user fee would further improve equity and promote use of long-term methods.

## Data Availability

The datasets generated and analysed during this study are in the possession of the authors and are available for sharing.

## Abbreviations

AIDS: Acquired immuno-deficiency syndrome
ACYP: Adjusted couple years protection
ANC: Ante-natal clinic
ART: Antiretroviral treatment
BP: Blood pressure/British pharmacopeia
BSN: Bachelor of Science in Nursing
CEA: Cost-effectiveness analysis
CHOICE: Choosing interventions that are cost-effective
CHW: Community Health Workers
CoC Pills: Combined Oral contraceptive pills
CPR: Contraceptives prevalence rate
CIP: Costed Implementation Plan
CSSD: Central Sterilization Services Department
CYP: Couple years protection
CWC: Children wellness clinic
DMPA: Depot medroxy-progesterone acetate
DHIS 2: District Health Information System 2
Eto: Etonogestrel
FP: Family planning
FY: Financial year
GBP: British Pound Sterling
GoK: Government of Kenya
HCP: Healthcare providers
HIV: Human immunodeficiency virus
IM: Intra-muscular
IMCI: Integrated management of childhood illness
IUCD: Intra-uterine copper device
IUD: Intra-uterine device
IUS: Intra-uterine systems
KDHS: Kenya Demographic and Health Survey
KEPH: Kenya Essential Package for Health
KeS: Kenya Shillings
KEMSA: Kenya Medical Supplies Authority
KHP: Kenya Health Policy 2012–2030
KHSSP: Kenya Health Sector Strategic and Investment Plan
KNBS: Kenya National Bureau of Statistics
KNH: Kenyatta National Hospital
LARC: Long-acting and reversible contraceptive
LNG—IUS: Levonorgestrel intra-uterine system
MCH: Maternal Child Health
MEDS: Mission for Essential Drugs and Supplies
PNC: Post natal care
PMTCT: Prevention of mother-to-child transmission
SD: Standard deviation
TB: Tuberculosis
UK: United Kingdom
UNFPA: United Nations Family Planning Agency
USA: United States of America
USAID: United States Agency for International Development
USD: United States Dollar
WHO: World Health Organization
